# Technical development and application of supercritical CO_2_ foaming technology in PCL foam production

**DOI:** 10.1038/s41598-024-57545-6

**Published:** 2024-03-21

**Authors:** Yujin Zhou, Yingrui Tian, Mengdong Zhang

**Affiliations:** 1 College of Physical Education, Wuhan Sports University, Wuhan, 430079 China; 2 College of Science, Wuhan University of Science and Technology, Wuhan, 430081 China; 3 School of Materials and Metallurgy, Wuhan University of Science and Technology, Wuhan, 430081 China; 4 Hubei Key Laboratory of Advanced Technology for Automotive Components & Hubei Collaborative Innovation Center for Automotive Components Technology, Wuhan University of Technology, Wuhan, 430070 China

**Keywords:** ScCO_2_ foaming, PCL, Molding technology, Forming methods, Combined technology, Application, Materials science, Materials for devices

## Abstract

Polycaprolactone (PCL) has the advantages of good biocompatibility, appropriate biodegradability, non-toxicity, flexibility, and processability. As a result, PCL-based foams can successfully work in bone tissue engineering, medical patches, drug delivery, reinforcing materials, and other applications. A promising technology for producing PCL foam products is supercritical CO_2_ (ScCO_2_) foaming technology, which avoids using organic solvents, is green, and has low foaming agent costs. However, due to the limitations of ScCO_2_ foaming technology, it is no longer possible to use this technology alone to meet current production requirements. Therefore, ScCO_2_ foaming technology must combine with other technologies to develop PCL foam products with better performance and matching requirements. This paper systematically reviews the technological development of ScCO_2_ foaming in producing PCL foams. The molding process of ScCO_2_ foaming and the conventional preparation process of PCL foam products are discussed comprehensively, including the preparation process, advantages, and disadvantages, challenges faced, etc. Six combined technologies for ScCO_2_ foaming in the production of PCL foams and the applications of PCL foams are presented. Finally, the future remaining research for producing PCL foams by ScCO_2_ foaming is analyzed.

## Introduction

Global fracture rates caused by an aging population, serious accidents, and other conditions such as bone disease remain high^1,2^. Globally, approximately 178 million new fractures and 455 million prevalent cases of acute or long-term symptoms of fractures were found in 2019, with a significant increase in the absolute count of fractures occurring^2^. Currently, an estimated 200 million people worldwide suffer from osteoporosis, which will lead to a continued increase in the incidence of fractures^3^. Fractures increase morbidity and mortality and, if left untreated, increase the risk of secondary fractures^3,4^ This can have a serious impact on the physical and mental health of the patient. As a result, tissue repair and replacement are of increasing interest^5^. Tissue engineering (TE) has become a promising area for regenerating and propagating damaged and dysfunctional tissues. Autologous bone grafting is often used for defective tissue repair, however, the slow recovery process of this treatment will result in prolonged hospital stays and high social health costs^6^. In addition, autologous bone grafts may suffer from a shortage of suppliers. Therefore, bone grafting in the form of a synthetic scaffold is a suitable alternative. In the field of tissue engineering, titanium alloys are used as bone grafting metals to repair bone defects. However, insufficient bioactivity is observed, and permanent implants may still be subject to erosion in vivo^7^. PCL is a bioresorbable polymer that is biocompatible and biodegradable, making it an ideal material for use in areas such as tissue engineering^8,9^. In addition, PCL has good mechanical stability and high durability in an aqueous environment, therefore, it is considered a viable material for the manufacture of scaffolds^5^. Polylactic acid (PLA) scaffold is also currently used in this field, a current research priority and biocompatible and biodegradable, and meets the basic biological criteria for tissue engineering applications^10^. However, PLA degrades at a significantly faster rate than PCL, but PCL scaffolds are more impact-resistant than PLA scaffolds, making PCL more attractive for long-term implants and controlled-release applications^11^. Due to these characteristics, PCL scaffolds can be used in bone implants to promote the production of mature biological tissue for the treatment of severe bone trauma^6^. However, in addition to the preparation of scaffolds, PCL-based polymer foams can also be used for medical patches^12^, drug delivery^13^, wall insulation^14^, etc. It can be seen that PCL foams have great application prospects.

**Figure 1:**
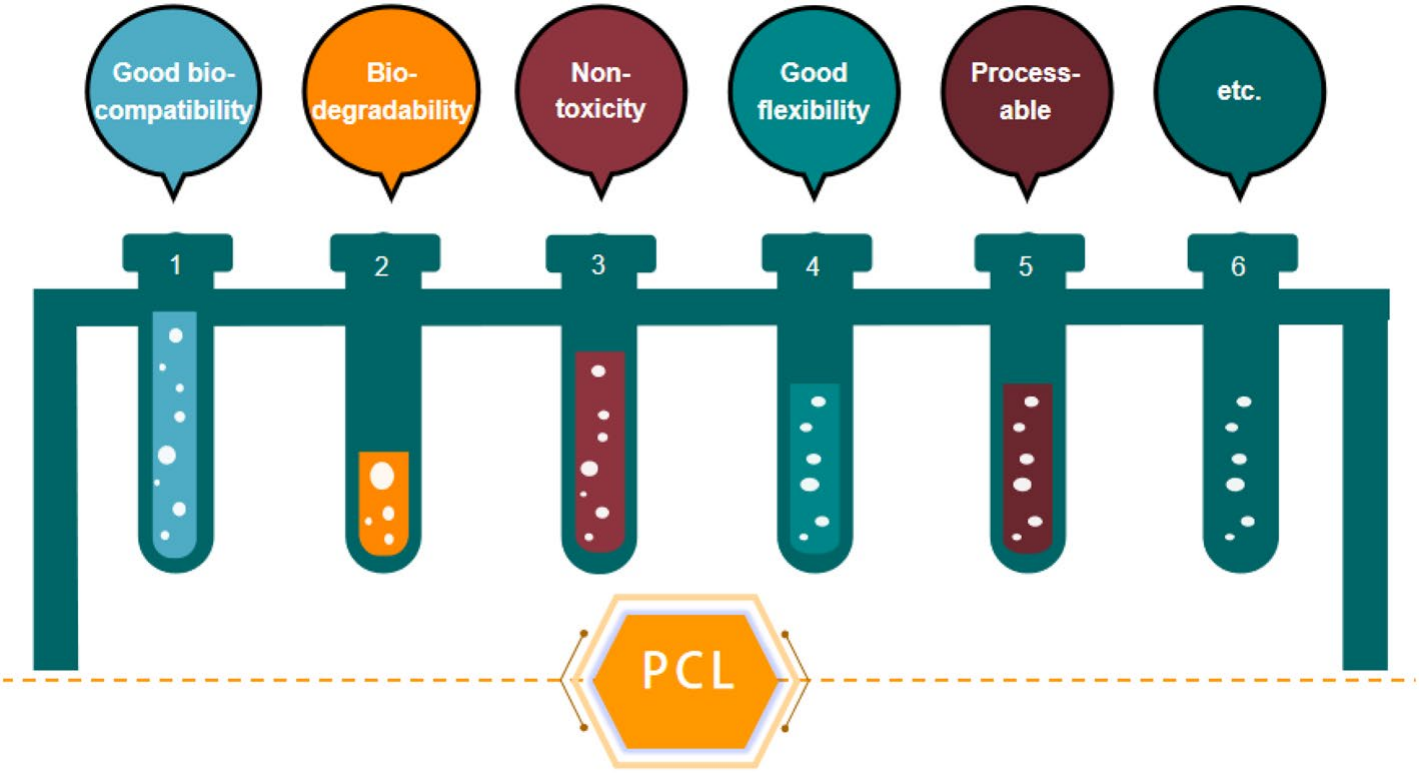
The main properties of PCL. This figure was created via WPS.

The advantages of PCL are shown in Fig. 1.

**Figure 2:**
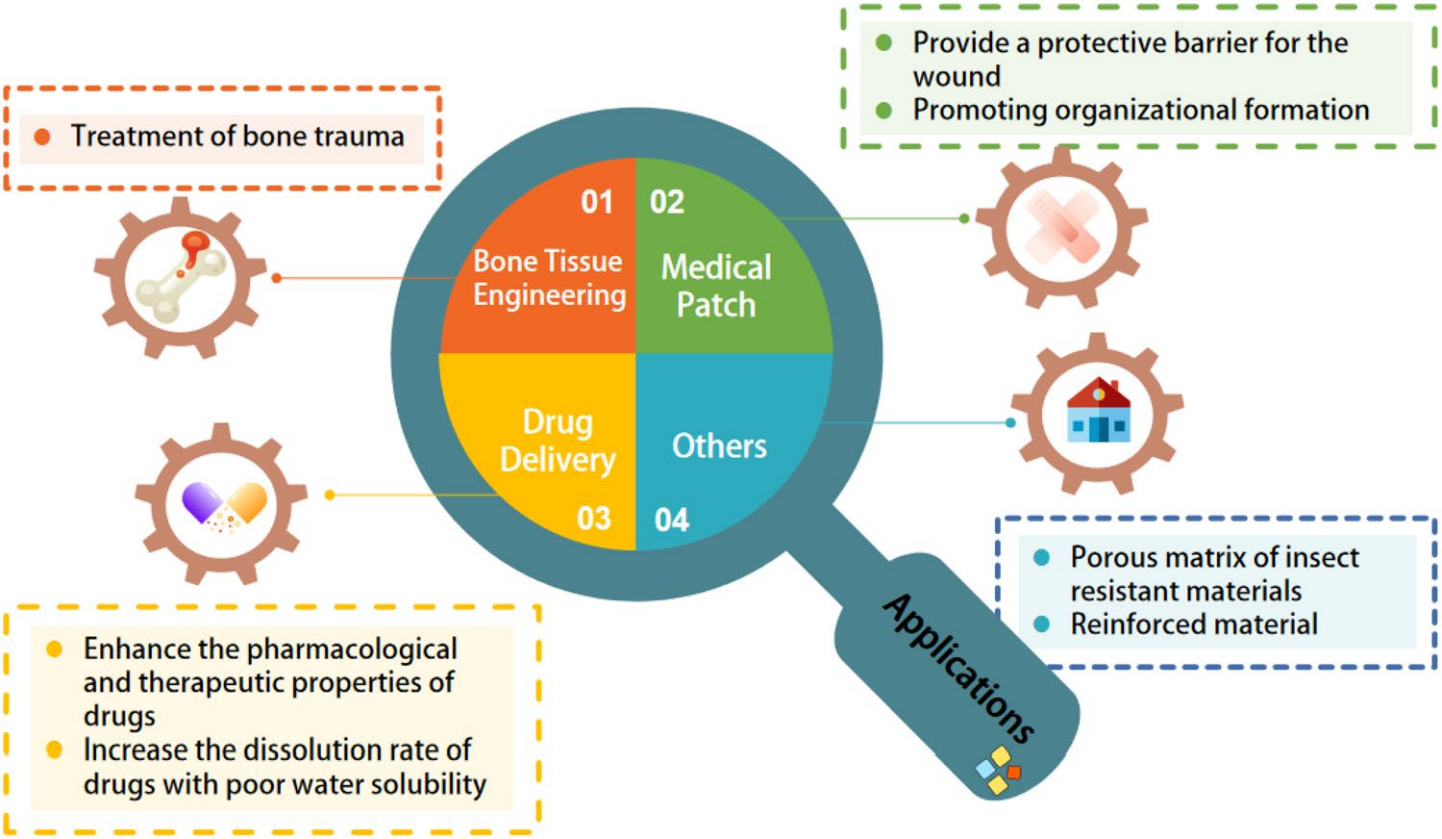
Applications of PCL porous materials in various fields. This figure was created via WPS.

PCL is an aliphatic polyester consisting of hexanoate repeating units widely used for biomedical applications and environmentally sustainable packaging. The controlled degradability, compatibility with other polymers, and the potential to be made from monomers of renewable origin are properties that make PCL a very useful polymer, but only if its properties can be controlled and its manufacturing costs are low^15,16^. PCL is biodegradable. It cleaves to short-chain alcohols and acids in contact with water and can be used as a porous matrix to develop sustainable and innovative materials^17^. PCL-based polymer foams are of great interest due to their excellent properties, non-toxicity, and low cost, and they are widely used in biomedical applications such as tissue scaffolds and drug delivery^18^. Figure 2 shows the current status of PCL porous materials in various fields of application. Here the applications of PCL foams in various fields are described in detail in categories.

### Bone tissue engineering

The high incidence of fractures due to an aging population, serious accidents, and other conditions such as bone disease has brought much attention to the repair of hard tissues^1^. Bone can repair itself under healthy conditions unless the degree of separation between bone fragments exceeds a certain threshold. When the separation of fragments exceeds a threshold, natural or synthetic grafts are required as a bridge to promote bone regeneration. However, autologous bone grafting has certain drawbacks, such as slow recovery, so grafting in the form of a synthetic scaffold is a promising method of treating severe bone trauma^6^. Porous scaffolds can mimic the extracellular matrix (ECM) and provide a template for cell growth and differentiation^19^. In this process, the pores of the scaffold play an important role. The pores allow cell migration and proliferation and are necessary for tissue formation and vascularization^20^. In addition, an ideal bone tissue engineering scaffold should also provide microchannels for the delivery of growth factors to accelerate the regeneration of damaged or defective tissues^21^. Most implant materials, from coronal vascular scaffolds to bone implants, are often made of ceramics, metals, and polymers. However, these implants have some disadvantages, such as ceramics being brittle and difficult to manufacture and metals being corrosive and toxic^7^. On the other hand, PCL is considered a suitable and promising scaffold material due to its advantages, such as good biodegradability and biocompatibility. More importantly, PCL is an FDA-approved polyester for both load-bearing and non-load-bearing tissue engineering applications^22^. Among the technologies accessible for preparing porous scaffolds with a similar structure to the bone, ScCO_2_ foaming has emerged as an attractive and renewable green technology^23^. Conventional production methods often require the use of high temperatures and/or organic solvents, and these processing conditions are often incompatible with drug scaffolds^24^. ScCO_2_ foaming technology allows scaffolds to be prepared under mild operating conditions while avoiding the use of organic solvents^6^. When foaming PCL, the foaming parameters (porosity, pore size, pore distribution, etc.) are strictly controlled. This is because, in the field of tissue engineering, the needs of the scaffold vary from site to site and from function to function of the implant. For example, as a bone implant, a highly functional scaffold should have a porosity of around 80%. Still, at the same time, a balance between degradation rate and mechanical properties should be maintained^25^. The ultimate goal of scaffolds in bone tissue engineering is to promote the production of mature biological tissue after a certain implantation period (approximately months or even years)^1^.

Although PCL has shown good properties for biomedical applications, it is a conductive polymer material. Whereas the application of electric fields can potentially enhance cell development and regeneration in various tissues, the addition of conductive materials such as graphene can broaden the application of PCL scaffolds^20^. PCL scaffolds should also contain bioactive agents, such as growth factors (GFs), to promote tissue growth^6^. Although various GFs have shown very positive effects, there are still limitations in terms of stability and the possibility of off-target or undesirable side effects^26^. In addition, the hydrophobic surface properties of PCL make it difficult for cells to adhere. In contrast, when chitosan (CS) is bound to PCL, the hydrophilicity of CS can modulate the hydrophobicity of PCL^19^.

### Medical patches

Medical patches are topical biomedical devices used to treat skin injuries and provide a protective barrier for the wound to ensure a good sterile environment for the wound healing process. At the same time, it promotes tissue formation due to the direct action of the drug^27^. PCL is the most promising wound-healing material among the various biocompatible polyesters^28^. Because the rheological properties of PCL at non-high temperatures make it easy to process PCL into different shapes^29^. ScCO_2_ foaming combined with impregnation is an innovative technique often used in the production of PCL porous patches. The use of ScCO_2_ acts as a solvent during the impregnation process. After the impregnation is complete, the ScCO_2_ can be removed directly under reduced pressure. The use of this technique overcomes the disadvantages of using organic solvents, which can affect the drug and are difficult to remove. However, attention should be paid to the pain caused during patch replacement at the wound site when producing the patch. Therefore, the development of innovative medical patches capable of prolonging the release of molecules that promote wound healing remains a focus of current research^28^. In addition, developing medical patches that can adapt their properties as the wound condition changes and are unique to each wound is a current challenge^28,29^.

### Drug delivery

Drug delivery systems (DDS) are designed to enhance the pharmacological and therapeutic properties of drugs or increase the dissolution rate of poorly water-soluble drugs^29^. The earliest designed DDS was based on nanoparticle-mediated delivery; however, nanoparticles are potentially cytotoxic, and therefore the design of biocompatible implants is a new chemotherapeutic strategy. On the other hand, PCL has a slow degradation rate and hydrophobicity, which can be used for long-term drug delivery^30^. PCL has been used in implants for long-term drug delivery and in patches for controlled drug delive^29^. In general, the bone scaffolds mentioned above also function as drug transporters. In addition, other implants can deliver drugs. For example, PCL artificial implants prepared by SFM can be used as an ophthalmic drug delivery system, overcoming the drawbacks of traditional intravitreal injections that may cause side effects such as inflammation, retinal detachment, and cataracts^29^. However, drug release efficiency is also one of the issues requiring attention in this field. Most drug delivery systems usually exhibit a poorly controlled first-order release, i.e., the release rate decreases over time. Suppose the dose released at the beginning of treatment is too high. In that case, potential toxicity can occur, while if the dose released is low to avoid toxicity, drug delivery is less efficient^31^.

### Others

PCL foam products are mostly used in the biomedical field, while there is little research on other applications. In addition to the above applications, PCL foam can be used as a porous matrix to load copaiba oleoresin to control the proliferation of Aedes aegypti larvae and insects, which is a promising application^17^. In addition, PCL can be used as a reinforcing filler to improve the water-resistance and other properties of the foam. For example, prepared PLA/PCL/RS foams can be used in wall insulation foams and are expected to replace traditional non-degradable insulation foams^14^. Overall, the application of PCL foams in other areas needs to be further expanded.

Several methods have been developed to fabricate porous structures, including solvent casting/particle leaching^32^, electrospinning^33^, freeze-drying^34^, and phase separation^35^. However, these methods have some limitations, such as the need to use organic solvents and the difficulty of removing them after manufacture, the high production temperatures that accelerate polymer degradation, and the long processing times which can lead to delamination of the active substance, thus affecting product performance^7,36^. Although solvent casting/particle leaching techniques can produce effectively interconnected pore scaffolds^32^, the relationship between particle (leaching agent) size and scaffold properties has not been clarified^37^. Electrospinning technology requires the regulation of many parameters during production, making it difficult to control the diameter of the fiber product^38^. In addition, electrospinning produces porous scaffolds with small internal pore sizes and dense fiber arrangements^39^. The freeze-drying technique is simple but takes too long, and the product's surface may develop crusts, protrusions, and cracks^40^. In contrast, the often-used thermally induced phase separation (TIPS) technique may cause irregularly shaped pores^41^. Although there are many disadvantages in using these traditional processes, such as the difficulty of controlling the pore structure, they also have their advantages, and these techniques are still in use today.

Supercritical foaming technology (Sc-) is a unique solvent-free foaming method, and, in addition, the plasticization effect of ScCO_2_ reduces the melting point of crystalline polymers as well as the processing temperature^1,36^. Therefore, ScCO_2_ foaming technology is a suitable alternative for producing PCL foams. ScCO_2_ foaming technology has many other advantages, such as CO_2_ being non-toxic, non-flammable, chemically inert, inexpensive, and easy to achieve in supercritical conditions (Tc = 31 °C, Pc = 7.38 MPa)^42,43^. This technique allows researchers to control the pore structure by controlling the processing conditions (mainly gas concentration, temperature, and pressure drop rate)^19^. Although modeling of pore formation during ScCO_2_ foaming is still challenging^24^, porogens have now been found to be the most suitable approach for additional macropore families with well-defined porosity and narrow pore size distribution^1^. So now, new strategies are needed to adjust the incorporation of solid porogen in ScCO_2_ foaming^1^.

The ScCO_2_ foaming technology consists of two main steps: (1) thorough and homogeneous mixing of CO_2_ gas in a supercritical state with the molten raw material at the target working temperature and pressure; (2) pressure reduction to produce many bubble nuclei^1^. ScCO_2_ foaming technology combines molding technology to produce products that meet expectations. Current molding technologies include compression molding^44^, injection molding^45^, extrusion^46^, and 3D printing^47^. These forming techniques allow for foam products with different characteristics to be obtained. Compression molding allows the production of sheet or plate foams with a special microporous structure^48^. Injection molding enables the production of foam products with complex three-dimensional geometry and high dimensional accuracy, with less shrinkage, higher dimensional stability, and lower weight^49^. Extrusion allows the manufacture of low-density polymer foams with continuous profiles and simple 2D geometries^50^. 3D printed molding technology enables complex geometries to be achieved without the need for templates and production-specific tools^51^. When using supercritical gas foaming in conjunction with different molding techniques, PCL foams of different shapes and properties can be obtained.

In recent years, supercritical foaming technology has become increasingly visible, and many studies have been carried out on this technology. This has led to significant technological advances in the production of ScCO_2_ foaming technology for PCL foams. However, production using ScCO_2_ foaming technology alone is no longer sufficient to meet practical needs, so ScCO_2_ foaming technology is often combined with other technologies in current production to break through technical barriers. The effective development of combined technologies is currently a hot topic of research. Examples are the combination of particle leaching^52^, mixing^29^, impregnation^29^, freeze-drying^30^, breath figures^53^, mould patterning^54^, etc. ScCO_2_ foaming technology is challenging to control the pore size and pore size distribution when producing, so combining it with particle leaching technology is an effective method^1^. ScCO_2_ foaming technology combined with impregnation allows for the production of polymer/drug composite systems, and using ScCO_2_ impregnation avoids using organic solvents^27^. However, the solubility of the impregnation in the solvent may be limited, which can be overcome by combining ScCO_2_ foaming technology with freeze-drying^30^. When using ScCO_2_ foaming technology, the product's surface often does not form pores, and a so-called " surface layer " appears^53^. In contrast, the porous structure of the exterior of the product is facilitated by combining breath figures^55^. Combining ScCO_2_ foaming technology with mould patterning allows the realization of porous scaffolds with ordered arrays of microchannels^54^. However, although the use of these combined technologies can compensate to some extent for the limitations of ScCO_2_ foaming itself and thus produce a foam product that meets the requirements, there are inevitably some drawbacks. For example, when binding particles for leaching, the porogenic agent may not be completely leached out and the leaching solvent used may have an impact on the product. In addition, research into binding technology is still insufficient.

Despite the advances in ScCO_2_ foaming technology for the production of PCL foams, there are some challenges. For example, when foaming polymer samples using this technique, one needs to be aware of the hydrophobicity of PCL, a characteristic that is detrimental in certain applications, such as PCL scaffolds. In addition, the product develops a so-called "surface layer". Although the formation of external pores can be facilitated to some extent by the respiration diagram technique, this problem has not yet been fully solved. In addition, the target product's pore size and distribution are difficult to predict and precisely control^1^. Besides the difficulty of effectively controlling the formation of bubble holes, the exploration of the combination technology of ScCO_2_ foaming, the improvement of the performance of PCL foam products, and the reduction of the cost of foaming technology also need to continue to advance. Although PCL is a promising material for the medical field, its high cost is still a problem that cannot be ignored. More importantly, while research into the use of supercritical foaming technology for the production of PCL foams is ongoing, the suitability of the products is not yet sufficient. Further research is still needed on how to solve these problems.

Our research focuses on the technological advances and applications of ScCO_2_ foaming technology in the production of PCL foams. First, we analyze the ScCO_2_ foaming technology in detail from the individual forming technologies. Then we compare the ScCO_2_ foaming technology with the previous foaming technology of PCL foams and analyze the technological progress of ScCO_2_ foaming technology in the production of PCL foams. Finally, we discuss the future research of ScCO_2_ foaming technology in PCL foam production.

## Supercritical CO_2_ foaming technology

**Figure 3:**
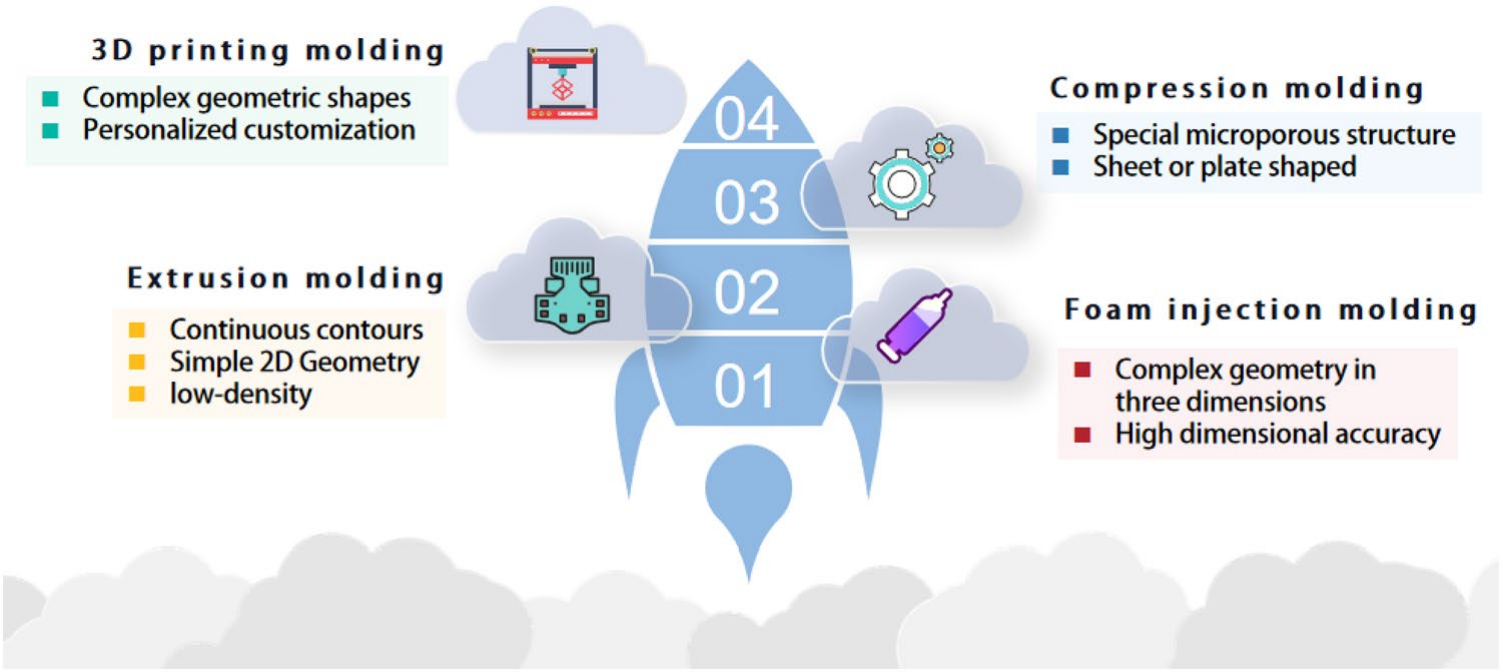
Forming process and characteristics of the corresponding product. This figure was created via WPS.

Supercritical foaming technology is a physical and microcellular foaming forming technology. Whether the technology is intermittent or continuous, the microcellular foaming process is divided into three processes: formation of a homogeneous gas/polymer system, cell nucleation, and cell growth^56^. Supercritical foaming technology needs to be used in conjunction with molding processes, including extrusion, injection molding, compression molding, and 3D printing. These four molding processes and the characteristics of the products produced are shown in Fig. 3. Supercritical foaming technology is increasingly interesting due to its solvent-free and environmentally friendly advantages. In this process, CO_2_ is widely used as a physical foaming agent due to its high solubility in polymers, non-toxicity, and low cost^57^. The forming process for ScCO_2_ foam technology is described in detail here.

### Foam injection molding

Injection Molding (IM) is a fast and versatile manufacturing technique used in the plastics industry to produce objects of different sizes, shapes, and details. It involves the injection of molten thermoplastic or thermoset materials into a closed mold under high pressure and temperature conditions. The finished product is cooled and/or solidified in the mold and removed at the end of the manufacturing cycle^58^. Foam injection molding (FIM) technology is an effective and cost-efficient method for producing foam products with complex three-dimensional geometry and high dimensional accuracy^49^. The basic process involves mixing polymer particles and a gas-foaming agent, melting them by heating, and injection molding them in a mold to form foam products. Foam injection molding processes offer less material use, more excellent dimensional stability, and faster production cycles^59^. In addition, products produced by the foam injection molding process have less product shrinkage, higher dimensional strength, lighter weight, better toughness, and higher acoustic and thermal insulation^37^. Micro-hole injection molding (MIM) technology is an advanced green foam injection molding technology that uses CO_2_ or N_2_ as a foaming agent to produce foam parts with complex structures^60^. This technology has great potential for the fabrication of porous polymer-based products^61^. MIM is achieved by conventional injection molding machines equipped with microcellular injection molding technology^62^. MIM has been widely used in many industries as a promising technology for manufacturing lightweight products due to its excellent flexibility and capabilities, good scalability and environmental benefits, low cost, high efficiency, and automated mass production^63,64^.

The microporous injection molding technology using supercritical fluids (SCF) as a foaming agent is known as the MuCell® process. This technology offers many advantages, such as material and energy savings, low cycle times, cost-effectiveness, and dimensional stability of the product^45^. MuCell® technology is commercially available and is the most widely used technology for manufacturing injection-molded foams. The MuCell® process was based on research developed at the Massachusetts Institute of Technology (MIT)^49^. The primary MuCell® process can be summarized in four main steps: mixing and dissolving the SCF in the polymer melt, cell nucleation, cell growth, and solidification^65^. Despite the advantages of the MuCell® process over conventional injection molding techniques, MuCell® also faces technical difficulties. Uncontrollable and non-uniform foam dimensions, poor surface appearance, and limited mechanical properties prevent the technology from being widely used^45,65,66^. A new microporous injection molding technology has emerged: IQ Foam® technology. The IQ Foam® technology is a technique used to produce engineering plastic foam products with excellent performance by controlling the flow of foaming gas to achieve different degrees of foaming in different parts. It is an intelligent processing technology that can adjust the foam structure to achieve different performance characteristics based on various design and application requirements. This technology can reduce the density of the products, and enhance their resistance to pressure, bending, and fatigue, while also improving surface quality and dimensional stability. The foam samples obtained by IQ Foam® technology have a thicker solid surface layer, lower cell density, and higher mechanical properties than MuCell® samples^67^. On the other hand, Coreback technology is a method used in the injection molding process to control foam structure by utilizing controllable counter pressure. This counter pressure can be reduced or eliminated during the mold-filling process, allowing for the formation of a more uniform and finer pore structure within the mold, resulting in lower density and improved performance. Compared to traditional injection molding techniques, using Coreback technology allows for the production of lighter, stronger, and stiffer foam products. This technology can be employed in the production of high-performance foam materials, such as lightweight structural components for automotive and aerospace applications.

Existing studies have shown that the mass of PCL parts increased when higher processing temperatures and injection flow rates were used during the injection molding process. When higher injection flow rates and lower processing temperatures were applied, the impact strength of PCL parts was more expressive, reaching values of around 260 J/m^68^. However, the mechanical properties of the PCL parts will vary depending on the injection molding method used. For example, the melt in MIM exhibits more excellent shear rates, temperature gradients, and faster cooling rates than conventional injection molding (CIM), allowing MIM products to show a unique morphology that differs from that of CIM products^69^.

### Extrusion molding

The basic principle of the extrusion process is to force a molten polymer through a die to configure, organize, and shape it^42^. ScCO_2_ extrusion foaming is a continuous plastic foaming technology that was initiated and developed by Park et al. and other researchers in 1990^46^. Unlike batch foaming processes, extrusion foaming has a unique feature in that the polymer melting, gas dispersion, foaming, and pelletizing processes are completed in a continuous process^70^. Extruded film/foam shows a good layer structure with clear layer boundaries and continuous film layers^71^. Such a continuous extrusion process is widely used on laboratory and industrial scales^72^. The continuous foaming process is more economical than batch foaming because it is easier to control and has a higher production capacity^42^. Extrusion foamings, such as capsules, spheres, and needles, can obtain various forms of bead foam^70^. Extruded bead blisters are more attractive than conventional autoclave bead blisters, not only in terms of significantly shorter production times, steps, and reduced energy consumption but also in terms of giving the beads a more controlled cellular structure^73^.

However, it can be challenging when considering the melt properties of the polymer, especially as extrusion foaming requires high melt strength^74^. Cross-linking is one of the most effective ways to improve the strength of polymer melts^46^. However, if over-crosslinked, the polymer may become a gel and cannot be used in the extrusion foaming process. It has now been proposed that dispersed micro/nanoparticles can act as nucleating agents to increase the cell density in extrusion foaming with little strain hardening reaction in the tensile flow of the blends^74^. This solution helps to solve the above problems, but the homogeneous dispersion of the nanoparticles is extremely challenging. In addition, strict control of factors such as melt pump, temperature distribution, and pressure reduction speed are also crucial for successful foam formation in the extrusion molding process. The melt pump used in extrusion molding is responsible for supplying plastic materials from hoppers or storage systems to the extruder. The melt pump needs to have a stable flow and pressure control capability to ensure a uniform plastic supply. Temperature is vital for the melting and flow of plastics in the extrusion process. Therefore, the extruder needs to have adjustable heating and cooling systems to achieve precise temperature control. Reasonable temperature distribution can ensure the uniform melting and flow of plastics, avoiding issues such as unstable melt or temperature control. Pressure changes during extrusion have a significant impact on the foam formation effect and foam quality. Typically, the extruder is equipped with pressure sensors and control systems to achieve accurate pressure control and reduction. By effectively controlling the rate and magnitude of pressure changes, a uniform foam structure, and stable foam formation effect can be achieved. In addition to the mentioned equipment requirements, several factors need attention in extrusion molding: Material selection: The material used in extrusion molding needs to have good meltability and flowability to ensure a smooth extrusion process and even plastic distribution. Different foam materials may require different processing temperatures and pressure conditions. Mold design: The mold used in extrusion molding needs to be designed appropriately based on the shape and size of the product to ensure uniform extrusion and shape retention of the plastic. The cooling system of the mold also needs to be carefully considered to avoid quality issues caused by overheating or overcooling. In summary, extrusion molding indeed requires key equipment and process requirements such as melt pumps, temperature distribution, and pressure reduction control in foam manufacturing. These requirements are crucial factors in ensuring the uniformity, stability, and consistency of plastic quality throughout the foam manufacturing process.

### Compression molding

Over the last few decades, compression molding has been one of the most widespread processes in the production of polymer parts. High automation, high geometrical accuracy, and fast cycle times are the main reasons for the proliferation of the process. The molding process does not require solvents, which makes the process necessary in the pharmaceutical sector, where solvent residues in the final product must be reduced^27^. The general process of compression molding: the preheated polymer is first placed into a heated mold cavity, and mechanical pressure is applied to the mold to inhibit the expansion of the compound^75^. That is, until the final release, the volume of the compound is essentially equal to the initial volume of the mold cavity. Finally, the compound expands immediately after the release of the mold clamping pressure. The resulting foam is cooled at room temperature. Compared to traditional chemical foaming agents, ScCO_2_ foaming by compression molding has a higher foaming multiplier and is more environmentally friendly. In foam technology, compression molding is commonly used for the production of foam plastic products such as Expanded Polystyrene (EPS) and Polyurethane (PU). EPS insulation boards are lightweight, high-strength thermal insulation materials widely used in residential, commercial, and industrial buildings. Compression molding involves adding EPS particles into a mold and then using heat and compression to expand them into foam and shape them into insulation boards within the mold. This process enables efficient and rapid production while ensuring product consistency and stability.PU cold storage panels are materials used in the construction of cold storage facilities, refrigerated warehouses, and other cold chain applications. They possess excellent insulation and corrosion resistance properties. Compression molding is employed to add PU particles into a mold and then use heat and compression to expand them into foam and shape them into cold storage panels within the mold. This process allows for efficient and rapid production while guaranteeing product consistency and stability. EPP packaging materials are lightweight and impact-resistant packaging materials widely used in electronics, automotive components, furniture, and other industries. Compression molding is utilized to add EPP particles into a mold and then use heat and compression to expand them into foam and shape them into packaging materials within the mold. This process enables efficient and rapid production while maintaining product consistency and stability. Compression molding technology finds extensive industrial applications in the field of foam plastics. It facilitates the production of various high-quality and high-performance foam products while achieving efficient and fast production processes, thereby increasing production efficiency and reducing manufacturing costs.

There are few descriptions of compression molding in the current literature, with most studies making direct use of the technique. An example is the preparation of micro-thick porous polymer sheets by ScCO_2_ compression molding foaming, an emerging technology^76^. Furthermore, introducing a high-temperature compression molding process in preparing PMVQ foams by ScCO_2_ foaming technology and post-vulcanization methods improves the vulcanization efficiency and makes the honeycomb structure more stable^44^. ScCO_2_ compression molding foaming combined with the impregnation process to produce PCL medical patches^27^. However, compression molding of wet foams can be complex because of the potential for densification and destruction of the foam structure. But this can be improved by using special clamping plates and low clamping forces^77^.

### 3D printed molding

3D printing is the common name for additive manufacturing (AM), which was created in the 1980s to address the particular requirements of model creation and rapid prototyping (RP)^78^. 3D printing is an emerging and fast-growing technology that uses digital model files to construct objects by printing layer by layer using bondable materials such as powdered metal or plastic. There are many different technological classifications for 3D printing, such as fused deposition modeling (FDM), electron beam free-form manufacturing (EBF), selective laser sintering (SLS), digital light processing (DLP), and many more. 3D printing technology eliminates tooling or machining (typical of traditional molding manufacturing) and allows custom parts to be made from metals, ceramics, and polymers^78^. However 3D printing of polymeric compounds is mainly limited to the manufacture of solid parts, in which case combined foaming and 3D printing technologies could provide an excellent alternative to the foam manufacturing techniques currently in use (e.g., foam injection molding)^50^. There are several methods combining 3D printing and foam technology, including the temperature control method, dual-material printing method, post-processing method, and powder bed sintering method. The temperature control method utilizes materials with foaming agents during 3D printing, and when the material reaches a certain temperature, the foaming agent is activated to expand and form a foam structure. The dual-material printing method uses two different materials, one of which is a meltable material and the other is a foaming agent, to print the external structure of the model with the meltable material and then inject the foaming agent into the internal cavity to form a foam structure. The post-processing method applies specific foaming agents or chemicals to the printed model after 3D printing to penetrate the material and expand to form a foam structure. The powder bed sintering method applies to powder materials such as metal or ceramics. The powder material is first 3D printed into a solid structure and then sintered at a specific temperature and atmosphere to form a foam structure through a solid-state diffusion reaction. Zhou^79^ et al. introduced a hierarchical porous polymer scaffold fabrication method. First, a polylactic acid (PLA) scaffold with large pores ranging from 100 to 800 μm was prepared using fused deposition modeling (FDM) technology. Then, gas foaming was used to form micro-pores of 1 to 10 μm in the scaffold. This combined technique avoids the disadvantages of pure 3D printing or gas foaming technology and generates positive synergistic effects, which can be used to manufacture layered porous scaffolds. The design of scaffold porosity provides an innovative opportunity to control cellular properties in a three-dimensional microenvironment.

3D printing technology is an automated method that can increase labor rates, cost efficiency, and material utilization, and improve manual safety^51,80^. The most striking feature of 3D printing technology is that it allows for individual customization and the production of precise and complex products. In the field of tissue engineering, the use of this Computer-Aided Tissue Engineering technology enables the printing of unique functional scaffold sheets in individualized shapes to address cartilage defects or to design scaffolds for complexly shaped human organs^81^. The combination of ScCO_2_ foaming technology with 3D printing technology is also a hotly researched direction at the moment. In addition to being used in tissue engineering, they can also be used in construction^51^, piezoelectric energy storage^82^, and many more. However, although porous materials of almost any geometry can be manufactured by 3D printing, the technique is time-consuming and requires complex and expensive equipment^83^.

## Conventional forming methods for PCL foams

**Figure 4:**
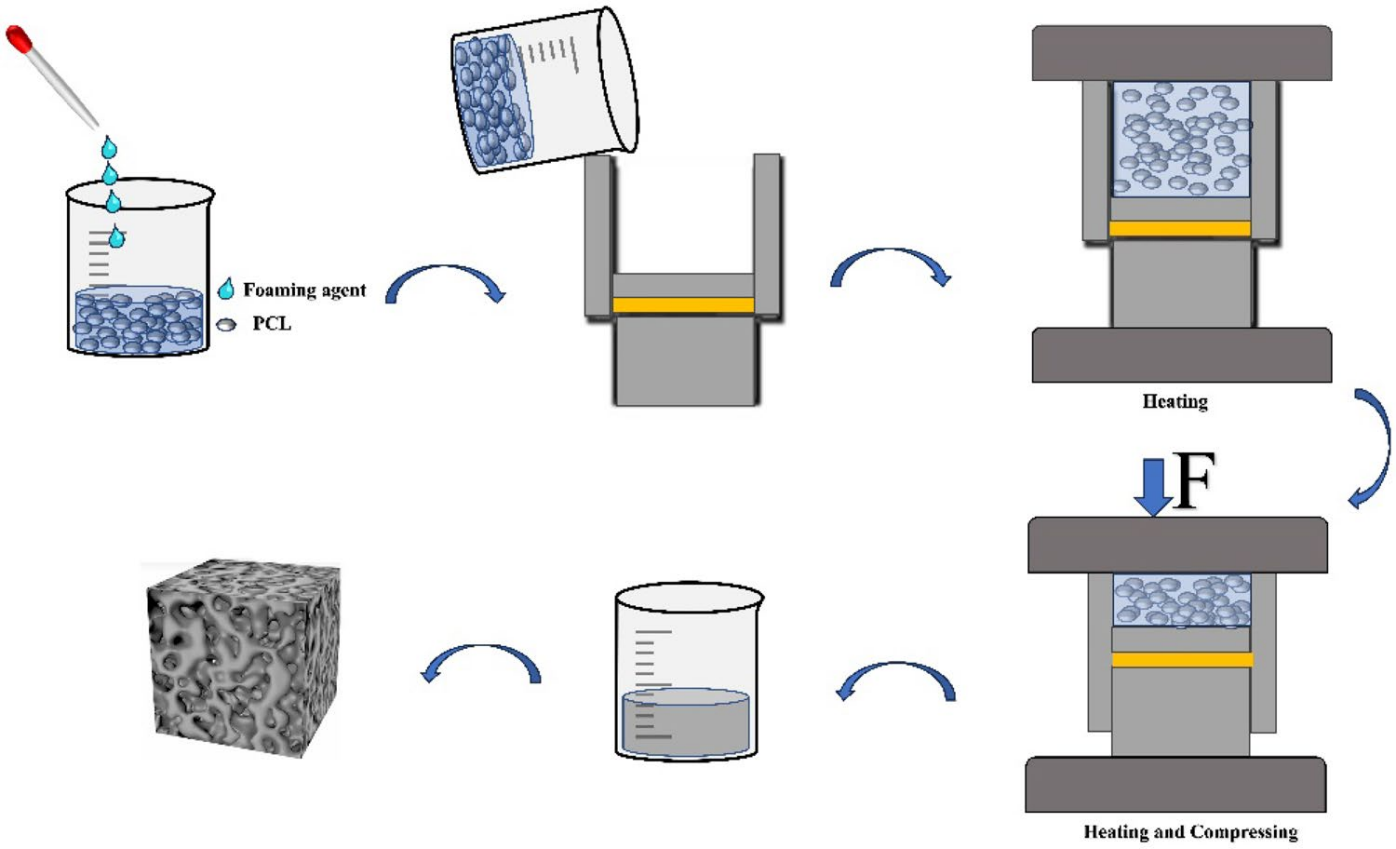
Schematic diagram of traditional PCL foaming methods. This figure was created via WPS.

PCL foams are currently used mainly in tissue engineering, and the traditional foaming and molding of PCL are examined here as examples of scaffolds. The main techniques are electrospinning, freeze-drying, phase separation, and solvent casting/particle leaching. Although electrospinning can prepare micron-sized polymer fibers, this technique makes it difficult to prepare complex 3D structures. Freeze-drying is a simple operational process, but the process is time-consuming and less productive. Thermally-induced phase separation, which is used more often, can produce fine porous structures, but the control of pore shape is slightly inadequate. Solution casting/particle leaching can effectively control the pore structure but faces the problem that the particles cannot be fully leached. When using these traditional processes to manufacture stents, there is limited ability to control the internal and external structure of the stent, including pore size, pore morphology, pore interconnectivity, and overall porosity^8^. In addition, they are not always suitable for all types of polymers. For example, thermally induced phase separation (TIPS) techniques are not ideal for producing thermoset foams due to the highly cross-linked network of thermoset resins^83^. However, these traditional processes are also advantageous and are still used in current research. The advantages and disadvantages of conventional production processes for PCL porous materials are shown in Table 1 (Fig. 4).

**Table 1:** Conventional production processes for PCL stents and their advantages and disadvantages.

Production technology	Advantages	Disadvantages	Ref.
Electrospinning	(1) Manufacture of micron-sized polymer fibers (2) 3D scaffolds with controlled porosity and pore size can be produced	(1) The need to modulate many parameters (2) Poor productivity and reproducibility (3) The scaffolds produced have the disadvantage of small internal pore sizes and dense fiber arrangements (4) Difficulty in producing fibers smaller than a few microns in diameter at full flow rates	^39^
Freeze-drying	(1) Hybrid macroporous bone scaffolds (pore size > 50 µm) with high openness (70%) and interconnected porosity can be obtained (2) Cost-effective (3) Simple process	(1) Time-consuming (2) Product surface may develop crusts, protrusions, and cracked surfaces	^87^
Thermally-induced phase separation	(1) The ability to produce membranes with a wide range of different properties (2) The ability to produce fine porous structures (3) The ability to produce large pores of several hundred microns	(1) Irregularly shaped pores are often induced (2) The prepared foamable particles may have poor foaming capacity	^92^
Solution casting/particle leaching	(1) The foam produced has good properties; (2) Effective interconnected pore supports can be produced (3) Enhanced control of porosity and pore size of PCL scaffolds	(1) The relationship between particle (leachate) size and scaffold properties has not been clearly analyzed (2) Difficulty in controlling the distribution of particles in the polymer solution (3) Particle residues	^32^

### Electrospinning

Electrospinning is a method used to manufacture tissue engineering scaffolds with high porosity and good biocompatibility^39^. Electrospinning is of interest due to its ability to create micron-sized polymer fibers^38^. The essential equipment for electrospinning consists of three main parts: the spinneret section, the high-voltage power supply, and the metal collector. The production process of electrospinning goes through the following three main stages: (1) the jetting starts at the top of the spinneret and forms a linear jet; (2) the bending and deformation, accompanied by the evaporation of the solvent to form circular and spiral trajectories to form solidified nanofibers; (3) the collection of nanofibers^84^. To produce PCL porous scaffolds, electrospinning is often combined with gas foaming. Such a process can be called the bubble electrospinning process. In the production of PCL porous scaffolds by this process, 2D sheet-like structured PCL nanofibrous are first prepared by electrospinning, and then the PCL nanofibrous are immersed in a methanol solution of sodium borohydride (NaBH_4_) form multilayer 3D scaffolds. Rapid hydrolysis of NaBH_4_ in methanol solution generates a large amount of H_2_, which causes the 2D sheet-like structure to swell to form 3D scaffolds^85^.

Although electrospinning can produce 3D scaffolds with controlled porosity and pore size^39^, the process still presents many challenges. Electrospinning requires regulating many parameters during production, making it particularly difficult to control the resulting fibers' diameter precisely^38^. Despite the simplicity of electrospinning in the laboratory, improving its productivity and reproducibility remains a great challenge^84^. In addition, scaffolds produced by the electrospinning process have the disadvantage of small internal pore sizes and dense fiber arrangements^39^, and it is challenging to manufacture fibers smaller than a few microns in diameter at full flow rates^38^. The electrospinning process still has many problems to solve on its way to meeting market demand.

### Freeze-drying

Freeze-drying, the full name is vacuum freeze drying. Freeze-drying consists of two main steps: (1) the material containing a large amount of water is cooled down in advance and frozen into a solid below the freezing point; (2) the ice is sublimated directly under vacuum conditions, thus removing the water for drying purposes. By such a method, porous structures can be produced. Freeze-drying is now widely used in the food sector, pharmaceuticals, etc., to dry and improve the stability of various products, including nanoparticles^86^. Freeze-drying is often used as an auxiliary process in conjunction with gas-foaming technology in the production of PCL foam scaffolds. The simultaneous application of freeze-drying and gas-foaming techniques allows the production of hybrid macroporous bone scaffolds (pore diameter > 50 µm) with high openness (70%) and interconnected porosity^87^. This is a cost-effective and relatively simple method and a potential option in soft tissue regeneration^88^. In addition to the drying effect, the freeze-drying process can be used to introduce other compounds into the micro-pores of the scaffold to improve its performance. For example, introducing chitosan nanofibers (CSNF) into the micropores of PCL scaffolds by freeze-drying can further improve the biocompatibility of PCL scaffolds^89^. Although freeze-drying is a simple and commonly used process, it is time-consuming and can take several days to dry at a time^40^. In addition, the preparation of products by the freeze-drying process may result in crusts, protrusions, and cracks on the product's surface due to factors such as temperature, concentration, and cooling rate.

### Phase separation

Common phase separation techniques used in the production of porous materials include Non-Solvent Induced Phase Separation (NIPS) and Thermally Induced Phase Separation (TIPS). NIPS is a common technology for the production of polymeric membranes and is capable of producing membranes with a wide range of different properties^90^. In the production of scaffolds, NIPS typically creates holes in the surface of the scaffold through a solvent-non-solvent exchange. Compared to NIPS, TIPS has fewer impact parameters and its use in the production of PCL foam scaffolds is more frequent^91^. TIPS is a technology that produces polymer particles and porous films. In TIPS of polymer solutions, there are three main processes: (1) dissolution of the polymer in the solvent at high temperatures to form a homogeneous solution; (2) cooling down and phase separation of the solution; and (3) precipitation to give polymer particles^92^. TIPS induces phase separation by the thermal energy's driving force, resulting in pores' formation. To produce fine porous structures with TIPS, the composites are often dissolved in pure solvents or solvent/non-solvent mixtures and stored at low temperatures^93^. TIPS technology can also be used in conjunction with gas foaming technology, and this combined technology can be used to produce large pores in the hundreds of micron range in chitosan scaffolds^41^. However, TIPS can often cause irregularly shaped pores^41^. In addition, foamable particles prepared by TIPS may have a poor foaming capacity^92^.

### Solution casting/particle leaching

Solution Casting/Particle Leaching (SCPL) consists of two technologies: solution casting technology and particle leaching technology. The solution casting technique for biocomposite scaffolds consists of two main steps: (1) dissolving the polymer in an organic solvent and casting the solution into a predefined 3D mold; (2) subsequently allowing the solvent to evaporate^18^. The primary function of solution casting is to cure the casting material to obtain a product similar to the original mold cavity. Particle leaching is an effective process for manufacturing highly interconnected porous structures. Particle leaching also consists of two main steps: (1) introduction of the particles into the polymer matrix and (2) solvent leaching to leave a porous structure. The structure is essentially the same as the shape of the original particles. Thus, porosity can be controlled by careful selection of particle size, geometry, and density^83^. In addition, foams produced by particle leaching have good properties such as high surface area, interconnected pores, and robust mechanical support^18^. The combination of solution casting and particle leaching allows effective interconnected hole supports to be produced^32^. The SPCL process involves casting the composite into a mold after the salt particles have been uniformly suspended in a polymer solution and evaporating the solvent under air or/and vacuum^93^. SCPL offers enhanced control over PCL scaffolds' porosity and pore size compared to most manufacturing processes^20^.

However, although researchers have analyzed the design and manufacture of porous scaffolds using various manufacturing techniques, the relationship between particle (leachate) size and scaffold properties has not been clearly analyzed to date^37^. In addition, while particle leaching techniques make it easy to control the pore structure, they are difficult to control the distribution of particles in the polymer solution and lead to problems with residual particles in the scaffold^94^. Therefore, researchers need to further analyze the relationship between particle size and scaffold performance and seek solutions to the issue of residual particles. Additionally, alternative methods for fabricating porous scaffolds can be explored to meet different requirements. For electrospinning, parameter adjustments can continue to be improved to enhance productivity and repeatability. In freeze-drying techniques, optimization of drying time and resolution of surface structural issues can be addressed. As for phase separation techniques, attempts can be made to improve methods for controlling pore shape. Overall, further research and improvement are still needed in precise control of pore structure, enhancing production efficiency, and optimizing performance in the fabrication of porous scaffolds.

## Development of ScCO_2_ foaming technology in PCL foam production

**Figure 5:**
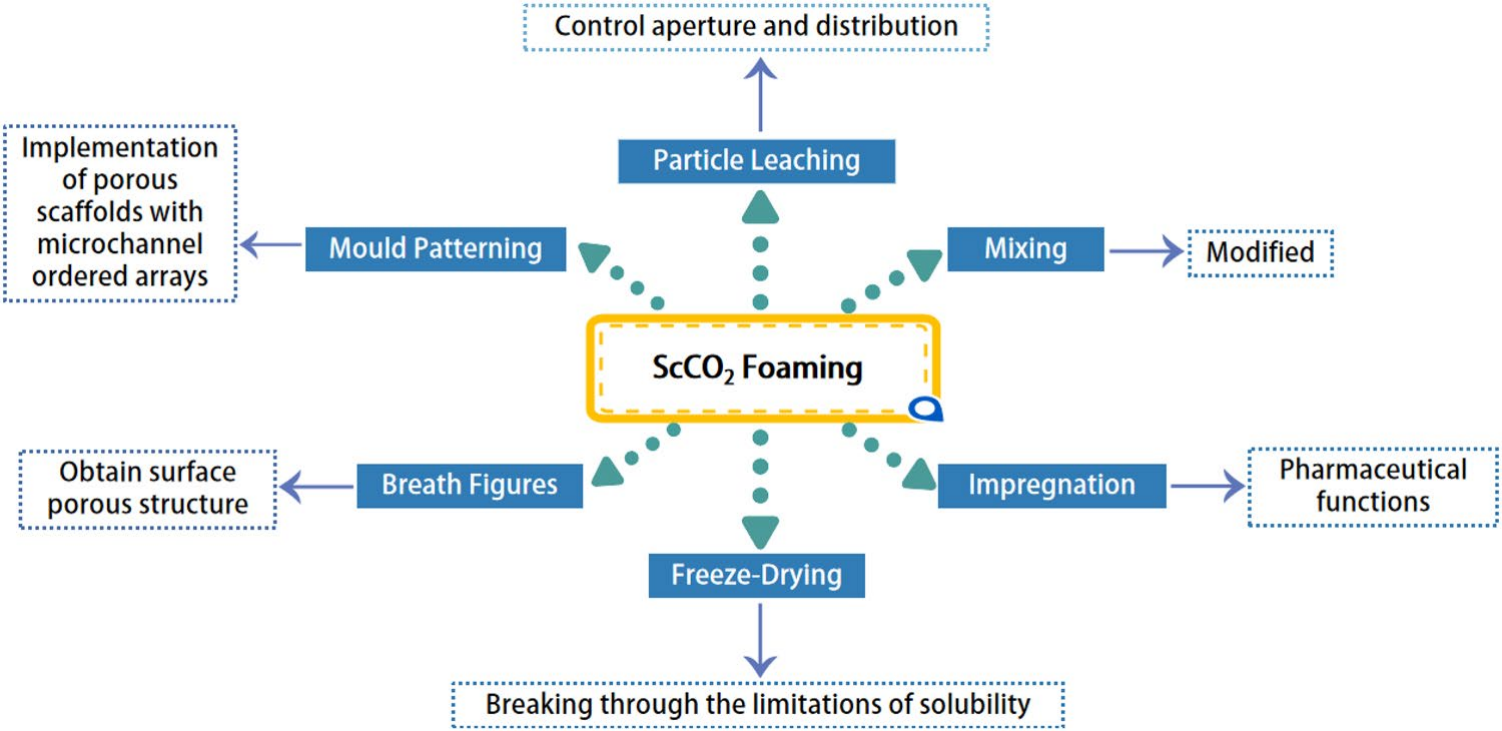
Combined technology for ScCO_2_ foaming and the effects it produces. This figure was created via WPS.

The methods described above are commonly used to produce PCL foam products. Although the research equipment is relatively simple and inexpensive to operate, it has many limitations^25^. For example, large quantities of harmful volatile organic solvents need to be used, or non-heat-resistant biological compounds cannot be used because of the high temperatures required. It's unfriendly to the human body and the environment. Supercritical foaming technology, on the other hand, effectively avoids these disadvantages. Supercritical foaming does not require the use of organic solvents and ScCO_2_ is the most commonly used supercritical fluid in the production of PCL foams. CO_2_ can easily reach the supercritical state, which means ScCO_2_ foaming does not require high-temperature conditions and avoids the thermal decomposition of organic compounds. Therefore, ScCO_2_ foaming technology can effectively incorporate bioactive materials into PCL foam products without changing their activity. Although the ScCO_2_ foaming strategy is a promising alternative for preparing PCL foams, preparing PCL foams with ideal porous morphology and stability by pure ScCO_2_ foaming is still a great challenge^36^. Figure 5 illustrates the combined technique for ScCO_2_ foaming and its effects. Here, the development of ScCO_2_ foaming technology for PCL foam production is examined in detail.

### ScCO_2_ foaming technology combined with particle leaching

ScCO_2_ foaming combined with the particle leaching technique is a method that utilizes supercritical carbon dioxide (ScCO_2_) as a foaming agent in conjunction with particle leaching. This technique can be applied in the production process of foam products in fields such as food, pharmaceuticals, cosmetics, etc., and it is known for its environmental friendliness and sustainability, thus receiving extensive research and application. Scholars in China have studied the process conditions for preparing potato starch foam materials using ScCO_2_ foaming combined with particle leaching technique. The results showed that optimized process conditions can yield potato starch foam materials with good physical and expansion properties. Additionally, researchers have investigated the application of ScCO_2_ foaming combined with particle leaching technique in the preparation of nanocellulose foam materials, and the results demonstrated that this technique can effectively improve the porosity and thermal stability of foam materials. In foreign studies, there have also been relevant research efforts. For example, an environmentally friendly corn starch foam material was prepared using ScCO_2_ foaming combined with a particle leaching technique, which exhibited good physical properties and thermal stability. Furthermore, researchers successfully prepared a novel protein foam material using ScCO_2_ foaming combined with particle leaching technique, which possessed excellent biocompatibility and degradability, showing promising applications in the field of medicine. Overall, ScCO_2_ foaming combined with particle leaching technique has made certain research progress in various fields. However, there are still some challenges, such as high production costs and the need for further improvement in the mechanical properties of foam materials. In the future, research in this field will continue to delve deeper to explore more efficient, environmentally friendly, and sustainable applications of ScCO_2_ foaming combined with particle leaching techniques.

**Figure 6:**
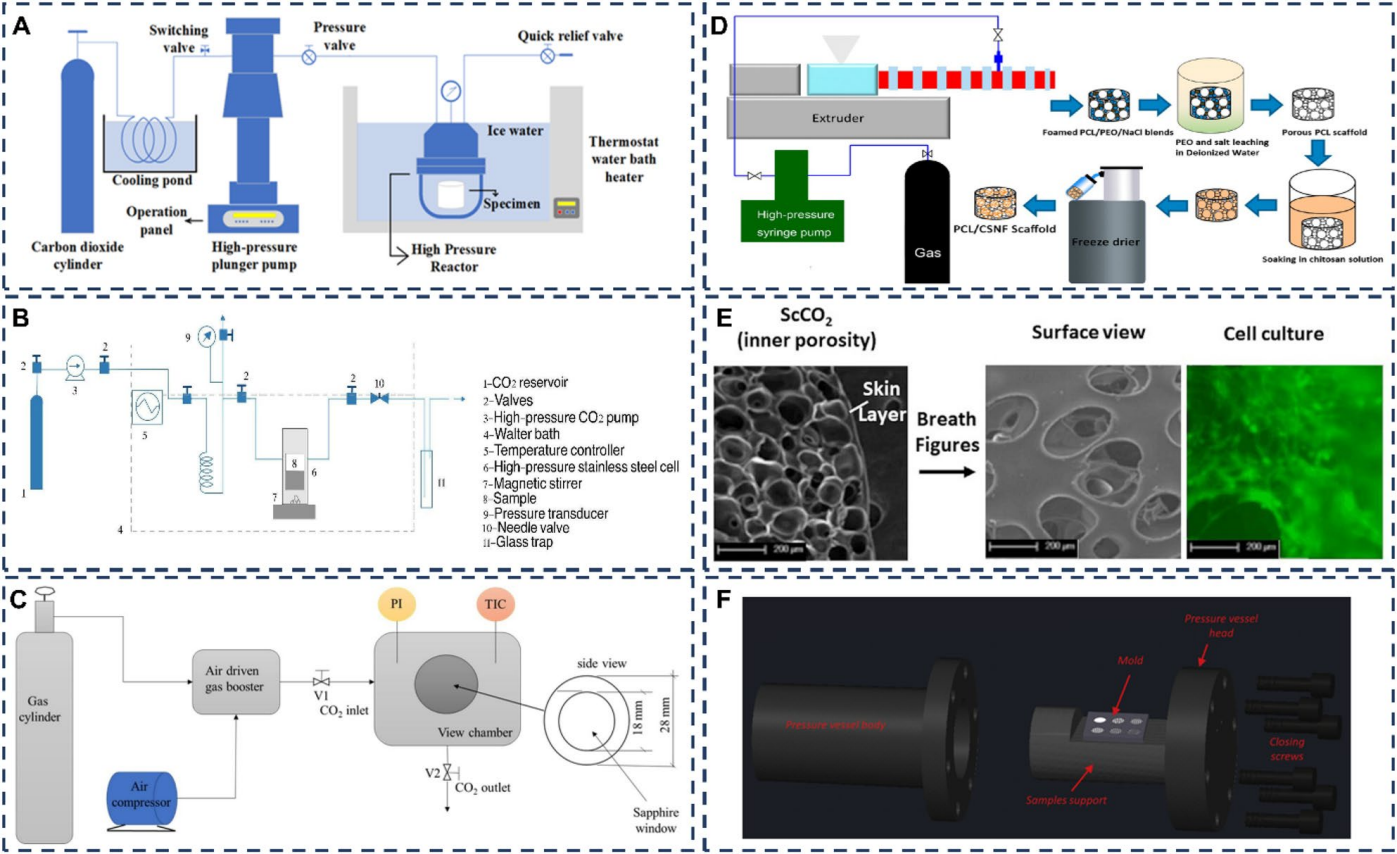
(**A**) ScCO_2_ foaming technology combined with particle leaching^50^, copyright © 2021 The Authors. (**B**) SFM technology^99^, copyright © 2013 Elsevier B.V. (**C**) ScCO_2_ foaming technology combined with impregnation^11^, copyright © 2022 The Authors. (**D**) ScCO_2_ foaming technology combined with freeze-drying^91^, copyright © 2014 American Chemical Society. (**E**) Bubble structure produced by ScCO_2_ foaming technology combined with BFs^57^, copyright © 2020 Elsevier B.V. (**F**) Core device for ScCO_2_ foam combined with patterning mould patterning^56^, copyright © 2019 Elsevier B.V.

ScCO_2_ foaming technology often makes it difficult to control the pore size and distribution when producing foams. To solve this problem, the most suitable method is to combine it with particle leaching techniques, i.e., the use of solid porogen. Figure 6A shows an apparatus diagram of the technology. By using porogen, macroporous families with well-defined porosity and narrow pore size distribution can be obtained^1^. The foams obtained by the leaching of bound particles are often open foams. However, very little literature has been devoted to studies comparing the effects of different porogens on foam formation.

Turng^95^ et al. prepared a high porosity and high connectivity porous scaffold by using a melt blending method to prepare a PLA/PEG/NaCl blend, followed by gas foaming and particle leaching processes. A self-made triple-screw composite extruder was used to improve the miscibility and dispersion of NaCl and PEG in the polylactic acid matrix. Supercritical carbon dioxide was used as a physical foaming agent for microporous foaming. Sodium chloride (NaCl) was used as a pore-forming agent to further increase the porosity of the PLA scaffold. The study investigated the effects of PEG and NaCl on the structure and properties of PLA-based blends, as well as the effects of the porosity, pore size, connectivity, and hydrophilicity of the porous scaffold. The results showed that the addition of polyethylene glycol and NaCl significantly increased the crystallization rate of polylactic acid and reduced its viscoelasticity. In addition, the scaffold prepared from PLA/PEG/NaCl blend had a bimodal interconnected porous structure with an open porosity of about 86% and a maximum porosity of 80%. The presence of polyethylene glycol in the PLA/NaCl composite material improved the extraction ability of NaCl particles during leaching, forming a good interconnect structure. The prepared porous scaffold was verified for biocompatibility by fibroblast culture for 10 days, with large variations observed.

Recently, K.K. et al. tested various types of porogen, including hydroxyapatite (HA), nitrocellulose (C), carboxymethylcellulose (CMC), and graphene oxide (GO) in the preparation of PCL foams by ScCO_2_ foaming technology^25^. By comparing the properties of ScCO_2_ foamed nHA/PCL, nC/PCL, and nGO/PCL composites with those of pure PCL plastic foams foamed under similar conditions, it was found that the addition of porogenic units led to a reduction in the pore size of the porous structures obtained. At the same time, the nucleation site density, mechanical strength, and crystallinity increased. The porosity of PCL foams obtained with these porogens exceeds 80%. In addition, the reduced pressure and saturation time result in a more uniform distribution of pores in the foam. In this test, the optimum conditions for producing biomedical PCL foams with the desired properties were 18 MPa pressure, 70 °C, and 1 h. The type and size of the porogen also had a significant effect on the properties of the foam. For example, the most significant but beneficial changes were obtained by adding graphene oxide and hydroxyapatite at the same concentration of the foaming process and porogen. For grains on the micron scale, an increase in concentration leads to increased pore size and decreased pore density. In contrast, for grains on the nanoscale, the opposite is true. The authors also propose a method for calculating porosity:

1 $$\uprho _{{{\text{foam}}}}  = \frac{{{\text{m}}_{{{\text{foam24}}}} }}{{{\text{V}}_{{{\text{foam}}}} }}$$

2 $$\upvarepsilon =\left(1-\frac{{\uprho }_{{\text{foam}}}}{{\uprho }_{{\text{PCL}}}}\right)\cdot 100\mathrm{\%}$$

where ρ_foam_ is the density of the solid foam (g/cm^3^), m_foam24_ is the mass of the sample after 24 h from the reactor (g), V_foam_ is the volume of the sample (cm^3^), and ε is the porosity. The density of PCL at room temperature is ρ_PCL_ = 1.145 g/cm^3^.

In addition, Burin et al. obtained insect-resistant porous materials by blending Copaiba (Co) oleoresin into PCL by the ScCO_2_ foaming/mixing method (SFM) using Pluronic F-68 (Plu) as a porogen^17^. Plu was blended into the whole material to form a porous structure while facilitating the release of hydrophobic Copaiba oleoresin in water. The porosity of the PCL: Plu (95:5) samples varied between 55 and 79%.

However, the most commonly used porogen are sugar or salt crystals because they have an extensive pore size distribution. However, removing this porogen is time-consuming, and removing the residues from the closed pores is almost impossible^96^. During the production process, the porogen often needs to be leached out using a solvent. This prevents the solvent-free nature of supercritical foaming technology from being exploited. In addition, during the leaching process, useful components may be leached out together, thus reducing the performance of the product. However, Rosales et al. obtained PCL drug scaffolds using the ScCO_2_ foaming technique and removing the porogen without the solvent-leaching process^1^. The porogen used by the authors is ammonium bicarbonate, which can be removed by thermal degradation at temperatures as low as 35–40 °C, which is well below the normal melting point of PCL. However, the amount of porogen will affect the final properties of the material. Therefore, the amount of porogen should be controlled. The results show that the maximum possible content of ammonium bicarbonate is set at 75% during the production of PCL scaffolds and that higher porogenic content leads to increased brittleness of the porous material. In addition to the mechanical properties of the product, the porogen also has an impact on the functionality of the product. For example, scaffolds prepared using ScCO_2_ foaming with ammonium bicarbonate porogen's have faster drug release kinetics due to the higher porosity and larger pore size of the scaffold after porogenic treatment, which facilitates the accessibility of aqueous media throughout the porous structure of the scaffold, thereby accelerating drug release^1^. Nevertheless, the leaching of the porogen is still an issue that needs to be addressed at present, as the strategy of using ammonium bicarbonate as a porogen does not apply to all areas of PCL foam production.

### Supercritical carbon dioxide (ScCO_2_) foaming/mixing method (SFM)

SFM is usually a method of mixing/blending additives into the foam material. A diagram of the experimental setup is shown in Fig. 6B. It has been frequently used in PCL foam production in recent years. However, detailed descriptions of this method are rarely covered in the current literature. The SFM method has been successfully applied to concentrate thermoplastic polymers, including PCL and other polyesters. By controlling the final depressurization rate and/or the amount of CO_2_ absorbed and varying other operating conditions (processing time, temperature, and pressure), materials with tunable physical properties and morphology, such as polymer crystallinity, porosity, pore size distribution, pore interconnectivity, and surface area, can be obtained^29^.

Supercritical CO_2_ (ScCO_2_) foaming/mixing is a technique that uses ScCO_2_ as both a foaming agent and solvent, combined with physical or chemical methods to process materials. This technology has gained widespread attention in the fields of materials science, chemical engineering, pharmaceuticals, and more due to its environmental friendliness, high efficiency, and lack of residue. There have been many domestic and foreign studies on ScCO_2_ foaming/mixing. Domestic research has mainly focused on areas such as food, medicine, and nanomaterials. For example, researchers have studied the application of ScCO_2_ foaming/mixing in vegetable lactic acid drinks, demonstrating that this technology can improve the quality and taste of the beverage. Additionally, researchers have used ScCO_2_ foaming/mixing to prepare a jasmine tea powder with good antioxidant properties while also investigating the effect of preparation process parameters on tea powder properties. Foreign research directions are more extensive, covering multiple fields such as materials, energy, and chemistry. For example, researchers have used ScCO_2_ foaming/mixing to prepare a polystyrene foam with excellent thermal stability and low density, which can be used for applications such as insulation and sound absorption. Additionally, researchers have used ScCO_2_ foaming/mixing to prepare a high-performance nanocomposite material for batteries. ScCO_2_ foaming/mixing is expected to find more applications in various fields.

Matos et al. successfully prepared dexamethasone (DXMT)-loaded polycaprolactone/silica nanoparticle (PCL/SNP) composites suitable for bone regeneration using the SFM method^97^. In this experiment, DXMT was first loaded into SNPs by ScCO_2_-assisted impregnation/deposition (SSID) and then doped into PCL by the SFM method. This study found that SFM treatment appeared to reduce the melting temperature of PCL, particularly at higher processing pressures (25 MPa), suggesting that the process used reduced the crystallinity of PCL. Burin et al. succeeded in incorporating copaiba oleoresin (Co) into PCL via SFM to obtain materials capable of controlling the proliferation of Aedes aegypti larvae and insects and avoiding mosquito transmission^17^. The authors propose that the efficiency of SFM depends on the components' thermodynamic and mass transfer properties and the physicochemical interactions of the pseudo-ternary systems (bioactive agent + ScCO_2_, bioactive agent + polymer, and ScCO_2_ + polymer). Raquel Boia et al. successfully developed a new PCL-based porous artificial implant with a highly porous structure that can be designed for better control of drug release by the SFM method^29^. The SFM method has a relatively important role to some extent in giving PCL foams the properties of an admixture on the one hand and in changing the physical properties of the bubble pores on the other. However, there are gaps in the current literature about its definition, principle, and process description.

In the future, research will focus on expanding the applications of ScCO2 foaming/mixing, optimizing the process, and improving its environmental performance. Additionally, many technical challenges need to be overcome in ScCO2 foaming/mixing, such as how to control foam size and distribution, and how to reduce production costs. Further research and development are required to address these issues.

### ScCO_2_ foaming technology combined with impregnation

Impregnation combined with ScCO_2_ foaming technology is a method that utilizes supercritical carbon dioxide (ScCO_2_) as a foaming agent and processes materials after impregnation. This technique has gained widespread attention in the fields of materials science, food, pharmaceuticals, and more due to its environmental friendliness, high efficiency, and low-cost advantages.

The combination of ScCO_2_ foaming and impregnation is an innovative technique for the production of polymer/drug complex systems, whereby ScCO_2_ allows the active ingredient to be impregnated into a polymeric porous matrix^27^. The schematic diagram of the device is shown in Fig. 6C. During the impregnation process, ScCO_2_ dissolves the active ingredient to be impregnated due to its high solvent capacity. In addition, it acts as a plasticizer and swelling agent for polymers, penetrating rapidly into the matrix and forming porous structures^28^. The technique of impregnation using ScCO_2_ is known as supercritical impregnation. The impregnation process can be divided into three steps: (1) dissolution of the active molecules in ScCO_2_ and contact with the impregnated matrix; (2) rapid diffusion and equilibration of the fluid phase within the pores of the matrix; (3) removal of ScCO_2_ under reduced pressure to obtain a solvent-free active compound-loaded matrix^29^. Impregnation is widely used in the pharmaceutical field. Traditional drug impregnation methods require organic solvents and thus have some disadvantages. Supercritical impregnation, on the other hand, overcomes the disadvantages of the traditional technique of using organic solvents and therefore results in a high-purity product^11,27^. Several studies have now demonstrated that supercritical foaming and impregnation occur in one step in the production process^27,29,98^. Both supercritical foaming and impregnation of PCL occur in an autoclave, which can also be divided into three processes: (1) the PCL and active material are first placed in a suitable position in the autoclave, then heated to a fixed temperature and slowly filled with ScCO_2_; (2) after reaching the working pressure, the system is kept for some time to ensure that the PCL foams, the active material is dissolved in ScCO_2_ and the fluid phase in the pores of the PCL foam is in diffusion equilibrium; (3) ScCO_2_ is removed from the vessel at a constant flow rate (approx. 1 MPa/min)^29^.

The combination of ScCO_2_ foaming and impregnation has been studied much in the production of PCL foam products. Alina Satpayeva et al. impregnated carvacrol into PCL scaffolds using ScCO_2_ foaming and impregnation techniques^11^. The excellent solubility of carvacrol in ScCO_2_ makes it an ideal candidate for designing new antimicrobial materials through the use of ScCO_2_. The results showed that the presence of carvacrol reduced the scaffolds' porosity, and the PCL scaffolds' impregnation yields were 7.22–7.71 wt%. In addition, the authors suggest that pressure reduction is the main factor controlling the impregnation yield and investigate the effect of different pressure reduction methods on impregnation yield, porosity, pore size, and morphology. Ignacio García-Casas et al. studied the foaming of PCL impregnated with quercetin using the ScCO_2_ batch foaming technique^98^. Quercetin is a flavonoid known for its antioxidant effects, and it also has anti-inflammatory, antibacterial, anticancer, and cardiovascular health-promoting properties. It was found that when the depressurization rate was low (0.1 MPa min^−1^), surfaces with large pores (~ 100 µm) and low cell number density appeared; when the depressurization rate was faster (20 MPa min^−1^), a large number of micropores and nanopores with higher cell number density appeared. The final PCL/quercetin composites with higher pore densities and smaller sizes were obtained at higher pressure and depressurization rates and lower temperatures (300 bar, 308 K, and 20 MPa min^−1^). PCL patches impregnated with nimesulide (NIME) were prepared by Roberta Campardelli et al. using ScCO_2_^29^. NIME is a non-steroidal anti-inflammatory drug that has good solubility in ScCO_2_. Studies have shown that NIME successfully adsorbs onto polymer foams when higher molecular weight PCL was used (H-PCL), whereas, in the case of lower molecular weight PCL (L-PCL), the maximum percentage by weight of NIME adsorbed onto the polymer was less than 1%. This is caused by the different shapes and sizes of the pores formed by the PCL in the two cases. Paola Franco et al. successfully attempted a one-off process of simultaneously foaming PCL and impregnating mesoglycan (MSG) into porous structures^27^. The results showed that MSG loading reached a maximum of 0.22 mg MSG/mg PCL when subjected to 17 MPa, 35 °C, and 24 h. This condition also ensured optimal foaming of PCL particles. The authors state that such an MSG/foamed PCL film system could provide wound protection and proper regeneration of the epithelium and also reduce the frequency of administration in long-term treatment to avoid associated side effects. Table 2 lists information about the four impregnations mentioned above.

**Table 2:** PCL impregnation types, effects, and suitable impregnation conditions.

Impregnants	Type	Effects	Suitable impregnation	Ref.
Carvacrol	Monoterpene organic compounds	Anti-inflammatory, antioxidant, anti-tumor, anti-mutagenic, anti-parasitic, antibacterial	Pressure: 20 MPa; Temperature: 40 °C; Impregnation time: 3 h; Decompression rate: 0.5 MPa/min (larger porosity can be obtained)	^11^
Quercetin	Flavonoids	Antioxidant, anti-inflammatory, antibacterial, anticancer, cardiovascular health	Pressure: 300 bar; Temperature: 308 K; Impregnation time: 1 h; Decompression rate: 20 MPa/min	^98^
Nimesulide	Non-steroidal anti-inflammatory drugs	Treatment of acute pain, inhibition of fungal growth, fungal prostaglandin E_2_ production, and enzyme activation	Pressure: 17 MPa; Temperature: 35 °C; Impregnation time: 24 h; Decompression rate: 1 MPa/min	^29^
Mesoglycan	Natural mucopolysaccharide preparations	Promotes wound healing	Pressure: 17 MPa; Temperature: 35 °C; Impregnation time: 24 h; Decompression rate: 0.1 MPa/min	^27^

ScCO_2_ foaming combined with impregnation is a promising technique, especially in the medical field. The above studies show that the depressurization rate is the key controlling factor for this binding technique, as it causes the nucleation and growth of the bubbles^99^. The current control of the decompression rate can be divided into two types: (1) one-step depressurization; and (2) two-step depressurization. One-step depressurization refers to the selection of a fixed depressurization rate for depressurization, while two-step depressurization refers to the selection of different depressurization rates in the early and late stages of depressurization, generally using slow depressurization in the early stages and fast depressurization in the late stages. The slow decompression in the early stages favors the formation of large pores, and the rapid decompression in the later stages favors the formation of small pores and further growth of existing pores. This two-step decompression design is suitable for the production of bi-modal and highly interconnected foams for tissue engineering scaffolds^11^. Current research on this technology is focused on the pharmaceutical neighborhood, where most of the PCL foam products produced by this combined technology are medical patches and tissue engineering scaffolds. At the same time, its applicability in other areas should be further considered. Furthermore, no literature has yet emerged comparing the effects and impact of different impregnations.

### ScCO_2_ foaming combined with freeze-drying

The production method of ScCO_2_ foaming combined with impregnation is described above. However, this method has its limitation—the solubility of the impregnate in the ScCO_2_ fluid. Many active agents have little or no solubility in ScCO_2_. One solution is to use a high decompression rate, where rapid depressurization can facilitate the incorporation of the active compound into the polymer. However, rapid depressurization may lead to unwanted structural changes within the polymer structure, such as cracking, and may also damage the material^100^. Also, the regulation of the decompression rate is often complex and requires various factors to be considered. Aurelio Salerno et al. proposed an innovative solution to break the solubility limit by combining ScCO_2_ foaming and freeze-drying, an extremely attractive strategy in the field of pharmacology^30^. The authors propose that this method involves three steps: (1) preparation of crushed PCL pellet tablets from a concentrated solution of the drug in an organic solvent; (2) freeze-drying of the tablets to extract the organic solvent, followed by pre-compression to remove air bubbles; and (3) saturation and foaming of the tablets with ScCO_2_. A device diagram for this technique can be found in Fig. 6D. There have been many studies on the combination of ScCO_2_ foaming and freeze-drying technology both domestically and internationally. In domestic research, the focus has mainly been on the fields of food, pharmaceuticals, and nanomaterials. For example, researchers have used this technology to prepare a dietary fiber-rich black goji powder, demonstrating that the technique can retain the nutritional components and natural pigments of black goji. Additionally, researchers have developed a novel drug carrier with good drug-release properties using this technology. Internationally, there have also been significant advancements in the research of this technology. For instance, researchers have utilized ScCO_2_ foaming combined with freeze-drying to produce high-performance porous carbon materials with excellent adsorption and catalytic properties. Furthermore, researchers have used this technique to produce seaweed powder with good moisturizing and antioxidant properties. ScCO_2_ foaming combined with freeze-drying technology holds promise for applications in various fields. Future research will focus on expanding its applications, optimizing the process, and improving its environmental performance. Additionally, there are challenges to address, such as controlling the porosity and expansion properties of the materials, as well as optimizing process parameters to achieve the best results. Further research and development are needed to tackle these issues.

### ScCO_2_ foaming combined with breath figures (BFs)

Foam products obtained by ScCO_2_ foaming often have a "surface layer", which prevents the formation of external pores^53^. The formation of these surface layers has advantages and disadvantages, an advantage for the production of tight porous polymer components but a disadvantage for biomedicine, as such solid layers would prevent cell proliferation and the diffusion of nutrients and oxygen within the biomaterial^97^. The formation of surface layers is often difficult to avoid, and the combination of particle leaching can effectively obtain polymer foams with open pores on the surface. Furthermore, in 2018 Marta Castaño et al. proposed the production of PCL porous scaffolds with interconnected outer and inner pores by ScCO_2_ foaming combined with BFs^53^. The BFs technique is a method of forming porous surfaces through a simple process of solvent evaporation and water vapor condensation, which consists of three main processes: (1) deposition of the polymer solution on a solid carrier and its evaporation in a humid atmosphere; (2) during solvent evaporation, the solvent/air interface is cooled, and ambient water vapor begins to condense and form droplets on the solvent surface; and (3) micron-sized pores form on the surface as the condensed droplets evaporate^55^. In the production of PCL porous scaffolds by ScCO_2_ foaming combined with BFs technology, the porous structure inside the sample is first obtained by ScCO_2_ treatment, and then the porous structure on the surface is obtained by BFs technology. The microscopic bubble morphology of the product obtained by this technique is shown in Fig. 6E. It was shown that PCL porous scaffolds obtained by this binding technique performed well in both initial cell adhesion and biocompatibility, maintaining biocompatibility and improving cell behavior compared to untreated PCL porous scaffolds^53^.

The combined use of ScCO_2_ foaming and BFs technology is a relatively innovative production process with promising medical applications. However, this combined technology has been proposed in recent years and is still immature in its development. In-depth and comprehensive research on this technology is needed to put it to better use.

### ScCO_2_ foam combined with mould patterning

So far, ScCO_2_ foaming technical limitations have prevented the use of foaming alone to achieve porous scaffolds with ordered arrays of microchannels^45^. Aurelio Salerno et al. proposed a new method of mould patterning and combined it with ScCO_2_ foaming to obtain PCL supports^45^. Scaffolds produced by this method have an ordered array of microchannels. They are suitable for the regeneration of tissues with microtubular structures. The method developed by Aurelio Salerno et al. consists of three processes: (1) manufacture of a polytetrafluoroethylene (PTFE) mould with a predefined array of columns by a computer-aided machining process; (2) filling the PTFE mould with PCL followed by ScCO_2_ foaming; and (3) removal of the foam from the mould. The method combines patterned PTFE moulds, solution casting, and ScCO_2_ foaming. Figure 6F shows the core apparatus of the experiment. The authors suggest that uniform mould filling is an important prerequisite in this process, as the presence of voids and defects in the resulting PCL film will result in porous scaffolds with irregular structure and morphology.

## Future studies

Although the preparation of PCL foams using ScCO_2_ foaming technology overcomes many of the disadvantages of traditional preparation processes and is a highly promising green technology, some shortcomings in the production process need to be addressed. This section will discuss future remaining research on the current ScCO_2_ foaming technology in the production of PCL foams.

### Overcoming the hydrophobicity of PCL

PCL is hydrophobic, making it difficult for cells to adhere to the PCL scaffold. An ideal solution is to mix PCL with a hydrophilic substance such as chitosan when performing ScCO_2_ foaming. On the one hand, the hydrophobicity of PCL is adjusted, and on the other hand, the biocompatibility and permeability of the product are improved. However, the hydrophilic substances incorporated may not be biologically active enough, and it is necessary to introduce other structures to improve the activity. Hydroxyapatite (HA) nanostructure is a good choice, and HA can increase the bioactivity and osteoconductivity of the product. Unfortunately, the mechanical stability of HA is insufficient. In addition, when ScCO_2_ foaming is performed, the diffusion and adsorption of CO_2_ in the matrix decreases with the increase in the amount of HA incorporated, which leads to foaming difficulties^11^. Therefore, it is worthwhile to continue exploring overcoming the hydrophobicity problem of PCL and the foaming difficulties associated with the use of HA.

### Avoidance of "surface layer"

A prominent problem in the current production of PCL foams using ScCO_2_ foaming technology is forming the "surface layer". This "surface layer" is not conducive to forming external pores in the foam product. This will limit the range of applications for the product and there are already proposed solutions for this. Examples include the combination of ScCO_2_ foaming techniques with BFs techniques and/or particle leaching techniques, which produce surface porosity. Although these are the common means currently used and relatively good results have been achieved, this problem has not been completely solved. When using the particle leaching technique, there are inevitably problems associated with the choice of leaching solution and whether the salt-impregnated particles can be completely leached. Even if a porogenic agent such as ammonium bicarbonate, which can be removed by heating, is used, it needs to be ensured that it does not affect the activity of the thermosensitive bioactive agent. In addition, the use of ammonium bicarbonate causes the porosity and pore size of PCL scaffolds to increase, which in turn makes the scaffolds more brittle. When using the BFs technique, the size and distribution of the surface pores obtained may be difficult to control precisely. Therefore, the improvement and development of technology will continue to be required in the future.

### Exploration of hole formation mechanisms

It is often difficult to accurately control and predict the size and distribution of pore sizes when performing ScCO_2_ foaming, mainly because modeling the current pore formation mechanisms remains challenging. This will have many negative consequences. For example, the production of PCL scaffolds by ScCO_2_ foaming technology typically results in scaffolds with high macroporosity but low pore interconnectivity, which can impede cell entry and transport of nutrients and metabolic waste throughout the implant^26^. Although the size and distribution of pore size can be controlled by particle leaching, particle leaching may weaken the product's mechanical properties, such as tensile strength and impact resistance. The link between particle size and scaffold properties has not yet been clarified. Furthermore, the lack of a single characterization technique capable of fully elucidating the porous characteristics of the resulting scaffolds makes it difficult to select the optimal operating conditions for the defined scaffold structures^24^. Therefore, to obtain products with the required pore space, further research is needed on the mechanisms of pore formation.

### Development of ScCO_2_ foaming technology in combination with other technologies

Due to the limitations of ScCO_2_ foaming technology, it is difficult to obtain stable PCL foams with the desired porous morphology by using ScCO_2_ foaming technology alone^36^, so combining it with other technologies is often necessary. For example, technical limitations of ScCO_2_ foaming prevent the achievement of porous scaffolds featuring ordered arrays of microchannels through foaming technology alone, requiring a combination of mould patterning and solution casting to achieve this goal^54^. Depending on the actual requirements, the microstructure and properties of the foam are different, which requires a sufficiently large and well-developed body of technology. The combination of technologies mentioned above is insufficient, and some shortcomings need to be improved, so it is necessary to develop new technologies.

### PCL foam product performance enhancement

Although PCL has excellent properties such as degradability, processability, and biocompatibility and is a very important class of material in tissue engineering, it has some disadvantages that cannot be ignored. While the slow degradation rate of PCL makes it promising for long-term bone implants, different fields require different rates of degradation. Slow degradation rates, poor mechanical properties, and low cell adhesion are factors that largely limit the application of PCL foam products^21^. One solution is to blend PCL with other materials and foam them, thus achieving complementary properties. However, research summarising the relationship between complementary materials and performance enhancement is missing. Secondly, improving the surface uniformity of PCL foam products is also an issue to be addressed. For example, when using the foam injection molding process, poor mechanical properties and imperfect surface appearance can occur on the product's surface^101^. Surface inhomogeneities will have a significant impact on the performance of the foam product. The suitability of PCL scaffolds in the clinical environment is poor compared to ongoing experimental tests^5^. The environment within the human body is dynamic and complex and therefore the stability and antimicrobial properties of the stent need to be taken into account to avoid infection^102^. In addition, both stents and medical patches should have enhanced control of drug release and be able to provide continuous, prolonged drug delivery, allowing local drug concentrations to remain at effective levels for longer periods. In the next few years, antimicrobial resistance and controlled release are key issues to be addressed to improve the suitability of PCL stents.

### Cost reduction

The problem of the high cost of ScCO_2_ foaming technology has already been raised in a previous review^103^. However, the issue of the cost of the raw material PCL cannot be ignored. In 2022, the market price of PLA was US $3500–4100 per ton, while the market price of PCL was US $5900–6400 per ton (Source: HangHangCha). Compared to PLA, PCL is more costly. Therefore, PCL is often blended with PLA in the production of PCL porous materials to reduce production costs on the one hand and to improve the performance of the product on the other^104^. However, the current market price of PLA has increased significantly, mainly due to the introduction of the biodegradable plastics policy. This means that reducing costs by co-blending PLA with PCL may not be applicable in the future during the introduction of this policy. Therefore, efforts will need to be continued to develop suitable solutions in this area of cost reduction.

## Conclusion

**Figure 7:**
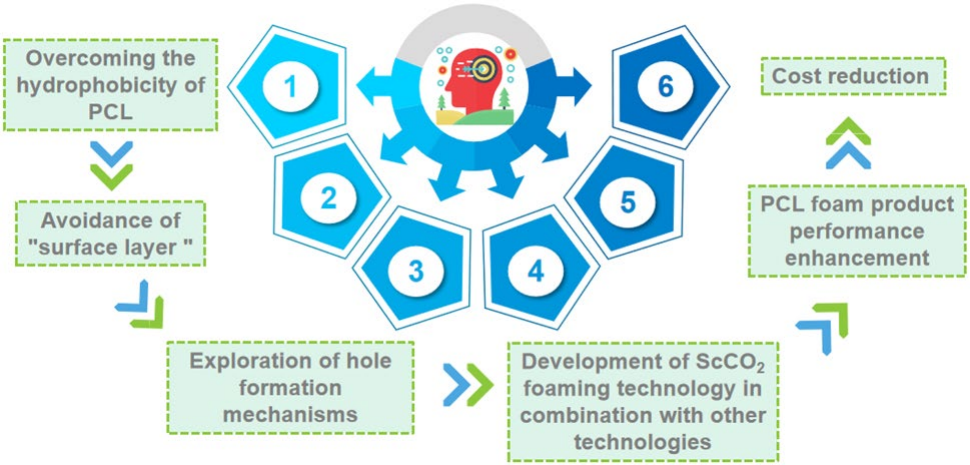
Future studies for the production of PCL foam products by ScCO_2_ foaming. This figure was created via WPS.

ScCO_2_ foaming is a green, solvent-free foaming technology that is ideal for the production of PCL foams. PCL foams are used in a wide range of applications, including tissue engineering, medical patches, drug delivery, and wall insulation foams. One of the most used applications is bone tissue engineering. As bone implants, PCL scaffolds are well suited for long-term implantation and controlled drug release due to their good biocompatibility, appropriate biodegradability, non-toxicity, high mechanical strength, good flexibility, and processability, providing the necessary sites for angiogenesis, new tissue formation, and remodeling. This will help to solve the challenge of human bone disease. To a certain extent, it can alleviate the heavy burden of bone diseases caused by the aging population and major accidents on families and society. PCL porous scaffolds are considered to be highly desirable green materials in the field of tissue engineering. Despite the significant advantages of using ScCO_2_ foaming technology to produce PCL foams, it is now difficult to produce products that meet practical requirements using ScCO_2_ foaming technology alone. An effective solution is to combine ScCO_2_ foaming technology with other technologies. The production of PCL porous materials is described in detail in terms of combining particle leaching, mixing, impregnation, freeze-drying, breath figures, and mould patterning. However, these technological developments are not sufficient and the process of producing PCL foam using ScCO_2_ foaming technology still faces many challenges, as shown in Fig. 7. To realize a wide range of product applications, the first step should be to overcome the shortcomings of the supercritical foaming technology and at the same time expand the technology to further enhance the performance of the PCL foam product. In addition, attention should be paid to cost reduction. PCL porous materials have great potential for medical applications, but if the cost issue is not solved, it will undoubtedly also be a financial burden for patients. In the future, PCL foam products, especially PCL stents, should also be customized to fit the patient's body condition. There is still a long way to go in the production of PCL foams through ScCO_2_ technology.

## Data Availability

Data is contained within the article.
